# Local differences in robustness to ocean acidification

**DOI:** 10.1242/bio.060479

**Published:** 2024-08-23

**Authors:** Dianna K. Padilla, Lisa Milke, Morodoluwa Akin-Fajiye, Maria Rosa, Dylan Redman, Alyssa Liguori, Allison Rugila, David Veilleux, Mark Dixon, David Charifson, Shannon L. Meseck

**Affiliations:** ^1^ Department of Ecology and Evolution, Stony Brook University, Stony Brook, NY 11794-5254, USA; ^2^ NOAA Fisheries Service, Milford Laboratory, 212 Rogers Ave, Milford, CT 06460, USA; ^3^ Department of Natural Resource Sciences, Thompson Rivers University, Kamloops, BC V2C 0C8, Canada

**Keywords:** Ocean acidification, Blue mussel, *Mytilus edulis*, Larval survivorship

## Abstract

Ocean acidification (OA) caused by increased atmospheric carbon dioxide is affecting marine systems globally and is more extreme in coastal waters. A wealth of research to determine how species will be affected by OA, now and in the future, is emerging. Most studies are discrete and generally do not include the full life cycle of animals. Studies that include the potential for adaptation responses of animals from areas with different environmental conditions and the most vulnerable life stages are needed. Therefore, we conducted experiments with the widely distributed blue mussel, *Mytilus edulis,* from populations regularly exposed to different OA conditions. Mussels experienced experimental conditions prior to spawning, through embryonic and larval development, both highly vulnerable stages. Survivorship to metamorphosis of larvae from all populations was negatively affected by extreme OA conditions (pH 7.3, Ω_ar,_ 0.39, *p*CO_2_ 2479.74), but, surprisingly, responses to mid OA (pH 7.6, Ω_ar_ 0.77, *p*CO_2_1167.13) and low OA (pH 7.9, Ω_ar_ 1.53, *p*CO_2_ 514.50) varied among populations. Two populations were robust and showed no effect of OA on survivorship in this range. One population displayed the expected negative effect on survivorship with increased OA. Unexpectedly, survivorship in the fourth population was highest under mid OA conditions. There were also significant differences in development time among populations that were unaffected by OA. These results suggest that adaptation to OA may already be present in some populations and emphasizes the importance of testing animals from different populations to see the potential for adaptation to OA.

## Teaser

**Summary:** Robustness to ocean acidification differs among populations of blue mussels; some are robust, some are not or may show local adaptation to changes in water chemistry. Development time also differed among populations but was unrelated to ocean acidification.

## INTRODUCTION

Ocean Acidification (OA) results from increased anthropogenic atmospheric CO_2_, which drives changes in the carbonate chemistry of ocean water, including the saturation state of aragonite (Ω_ar_) and corresponding pH (Salisbury et al., 2008; Breitburg et al., 2015; Feely et al., 2010; Gledhill et al., 2015). This change in carbonate chemistry has been demonstrated to affect calcified organisms, especially early life stages of bivalved molluscs (Andersson et al., 2008; Feely et al., 2010; Gazeau, et al., 2013; Waldbusser and Salisbury, 2014; Waldbusser et al., 2015; Siedlecki et al., 2021; Vargas et al., 2022). Increased monitoring of ocean and coastal water chemistry has revealed that coastal areas are already experiencing greater OA conditions (termed coastal acidification) than those projected for the oceans in the next 50–100 years (Ekstrom, et al., 2015; Hunt et al., 2022). Because changes in ocean chemistry are occurring at least 100 times more rapidly than any changes experienced over the past 100,000 years (Zeebe, 2012; Zeebe et al., 2016), understanding the ability of species to respond or adapt is critical (Coen et al., 2007; Cranford, et al., 2007; Cranford, 2019; van der Schatte Olivier et al., 2020). Prior OA work with marine calcifying animals has generally found large negative effects of OA on survivorship, primarily in early life stages such as larvae (Kleypas et al., 2006; Pörtner and Farrell, 2008; Melzner et al., 2009; Schoener, 2011; Kelly and Hofmann, 2013; Munday et al., 2013; Meseck et al., 2021). For bivalve larvae it appears that the saturation state of aragonite (Ω_ar_ <1.5), rather than pH per se, impairs shell building (Waldbusser et al., 2015). Effects of OA are, however, expected to be profound for a variety of physiological and developmental processes of many taxa (reviewed by Kroeker et al., 2013; Bindoff et al., 2019). We are just beginning to assess the variety of physiological and developmental processes that may be influenced by OA (Waldbusser and Salisbury, 2014; Somero et al., 2016), as well as the capacity of species to adapt to these changes. Thus far, it is apparent that not all species, or developmental stages within a species, are inhibited or negatively affected by OA conditions (Gooding et al., 2009; Gazeau et al., 2013). In some cases, differences among individuals within a given species have been found, indicating variation in the capacity to respond to OA (Gazeau et al., 2013). Most studies to date, however, have tested the effects of OA on only a single population or source stock of a species.

The number of species for which physiological experimental studies have been conducted to document changes in metabolism, growth, calcification, survival, and immune response across life-history stages in responses to OA continues to grow, especially for marine bivalves (Gazeau et al., 2013; Gledhill et al., 2015; Clements and Hunt, 2017). Most OA studies, however, are of short duration and include a single source population, making them unlikely to capture the full potential for response to OA conditions of a species across its geographic range. Using these data to predict long-term effects on species abundance (Cooley et al., 2015; Rheuban et al., 2018; Grear et al., 2020), is challenging at best. In addition, most models projecting the effects of OA do not include the potential for adaptation or an evolutionary response to OA conditions within species (e.g. Cooley et al., 2015; Grear et al., 2020). Thus, the potential for species to develop resilience to increased levels of OA (acclimation or adaptation), especially differences among populations from different locales, remains largely unexplored.

Understanding the capacity of animals to respond to climate-driven changes in local conditions is critical to evaluate the capacity of species to respond to environmental change in general, including OA (Dupont et al., 2010; Pistevos et al., 2011; Sunday et al., 2011; Grear et al., 2020). Recently, studies of blue mussels, *Mytilus edulis*, from populations periodically naturally enriched with CO_2_ found that their larvae had greater survivorship than those from populations without enrichment, showing short term selection and potential adaptation to CO_2_ (Thomsen et al., 2017), and suggesting the potential for local adaptation to OA.

To understand the long-term effects of OA on bivalves in coastal ecosystems (Reusch, 2014; Thomsen et al., 2017), we need to determine whether there is capacity within species to adapt to these environmental changes. In addition, it is important to determine whether there are differences in response to OA conditions among populations (Harvey et al., 2016; Wood et al., 2016; Thomsen et al., 2017) from areas with different environmental conditions. To address these questions, we focused on the *Mytilus edulis,* an important model species for studies of physiology and adaptation (Goldberg, 1975; Menge, 2000; Meseck et al., 2021). *Mytilus edulis* are common to most temperate Atlantic shores, and have been a model species for studying physiological responses under different environmental conditions for decades (Bayne and Newell, 1983). They are also used as sentinel organisms to monitor coastal water for pollution (Farrington et al., 2016; Beyer et al., 2017). Blue mussels are an important foundation species in both the eastern and western north Atlantic and prolific suspension feeder that occurs in very high densities that can represent up to 90% of the coastal benthic biomass (Enderlein and Wahl, 2004). Blue mussels provide numerous ecosystem services, including improving water quality and stabilizing shorelines, among others (Nielsen et al., 2016; Smaal et al., 2019). The blue mussel is also a commercially important species in Europe and North America, valued at $0.7 billion annually (US dollars, http://www.fao.org/fishery/culturedspecies/Mytilus_edulis/en). Recent years, however, have seen a decline in blue mussel densities in the northeastern USA; scientists have suggested that much of this decline has been directly or indirectly attributed to effects of climate change, including OA (Sorte et al., 2017).

**Fig. 1.**
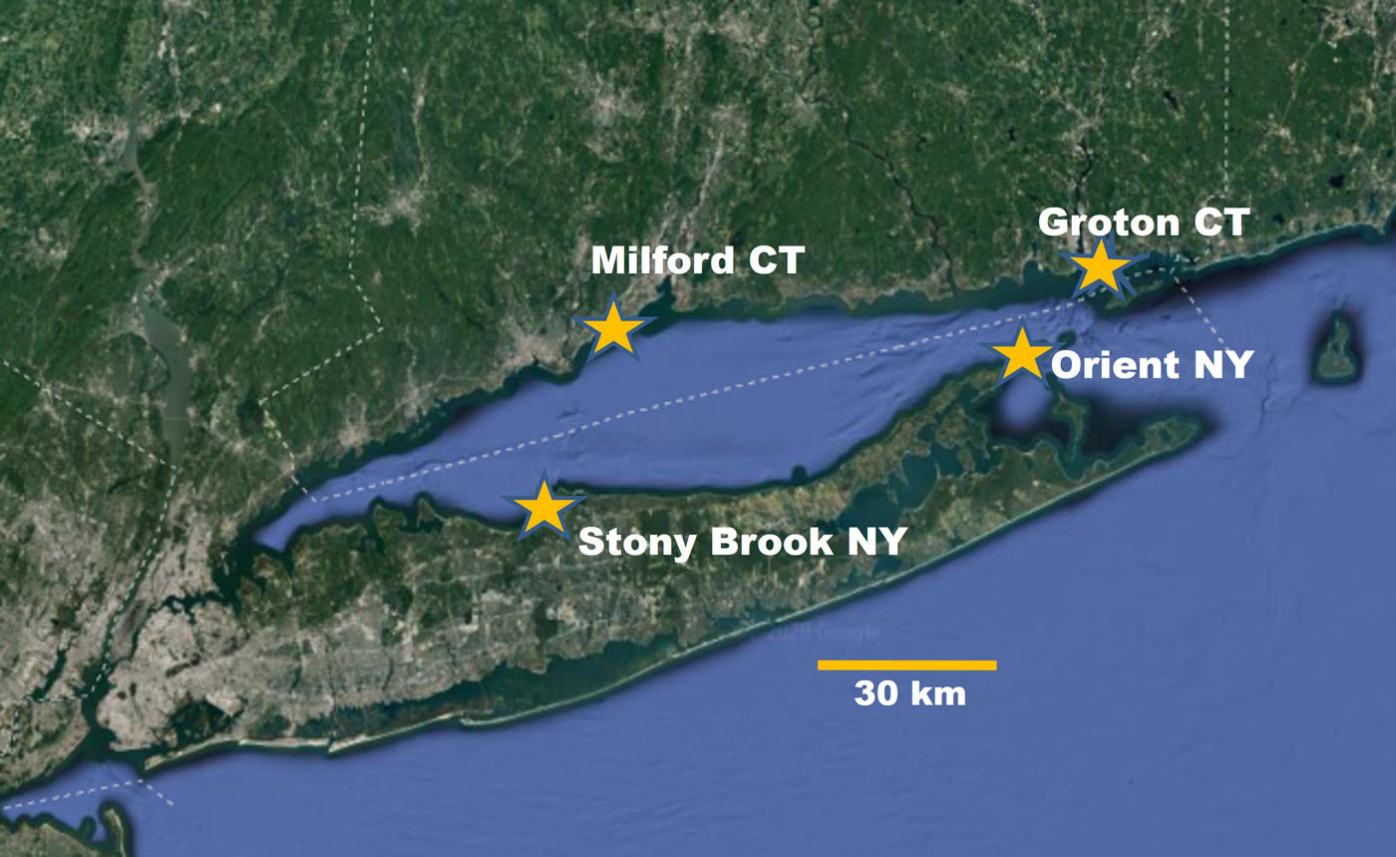
**Map of Long Island Sound and sites where blue mussels were collected (Google Earth image).** Milford CT, 41°12′35″N 73°03′03″W; Groton CT, 41°19′04″N 72°03′51″W, Orient NY 41°09′34″N 72°14′05″W; Stony Brook NY, 40°55′16″N 73°09′00″W.

To address the question of the potential for adaptation to three different OA conditions, we conducted controlled experiments with larvae through metamorphosis of blue mussels from four populations sampled from Long Island Sound (Fig. 1) sites that differ in local water conditions (Table 1).

**Table 1.**
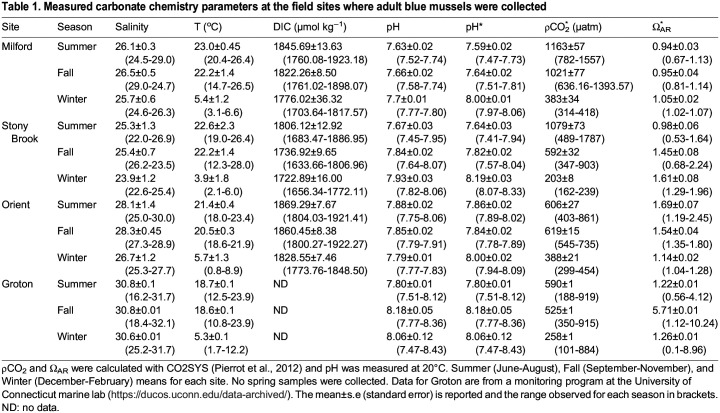
Measured carbonate chemistry parameters at the field sites where adult blue mussels were collected

## RESULTS

**Fig. 2.**
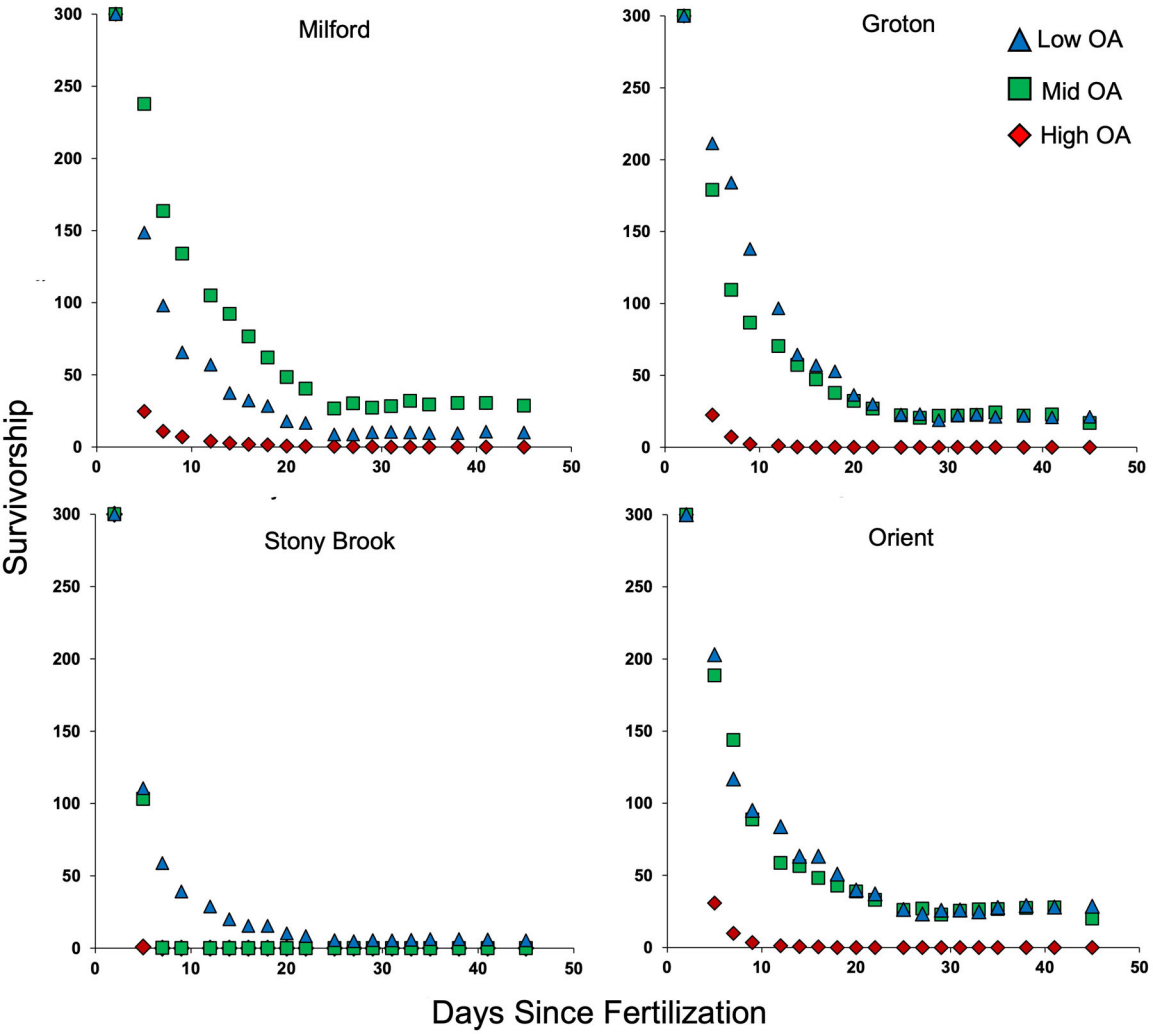
**Survivorship of larvae from each of the four test populations reared under each of the three OA conditions.** Shown are means of five replicates (error bars not included for clarity). Low OA (pH 7.9, Ω_ar_ 1.53, pCO_2_ 514.50), Mid OA (pH 7.57, Ω_ar_ 0.77, pCO_2_ 1167.13) High OA (pH 7.26, Ω_ar_ 0.39, pCO_2_ 2479.74).

**Fig. 3.**
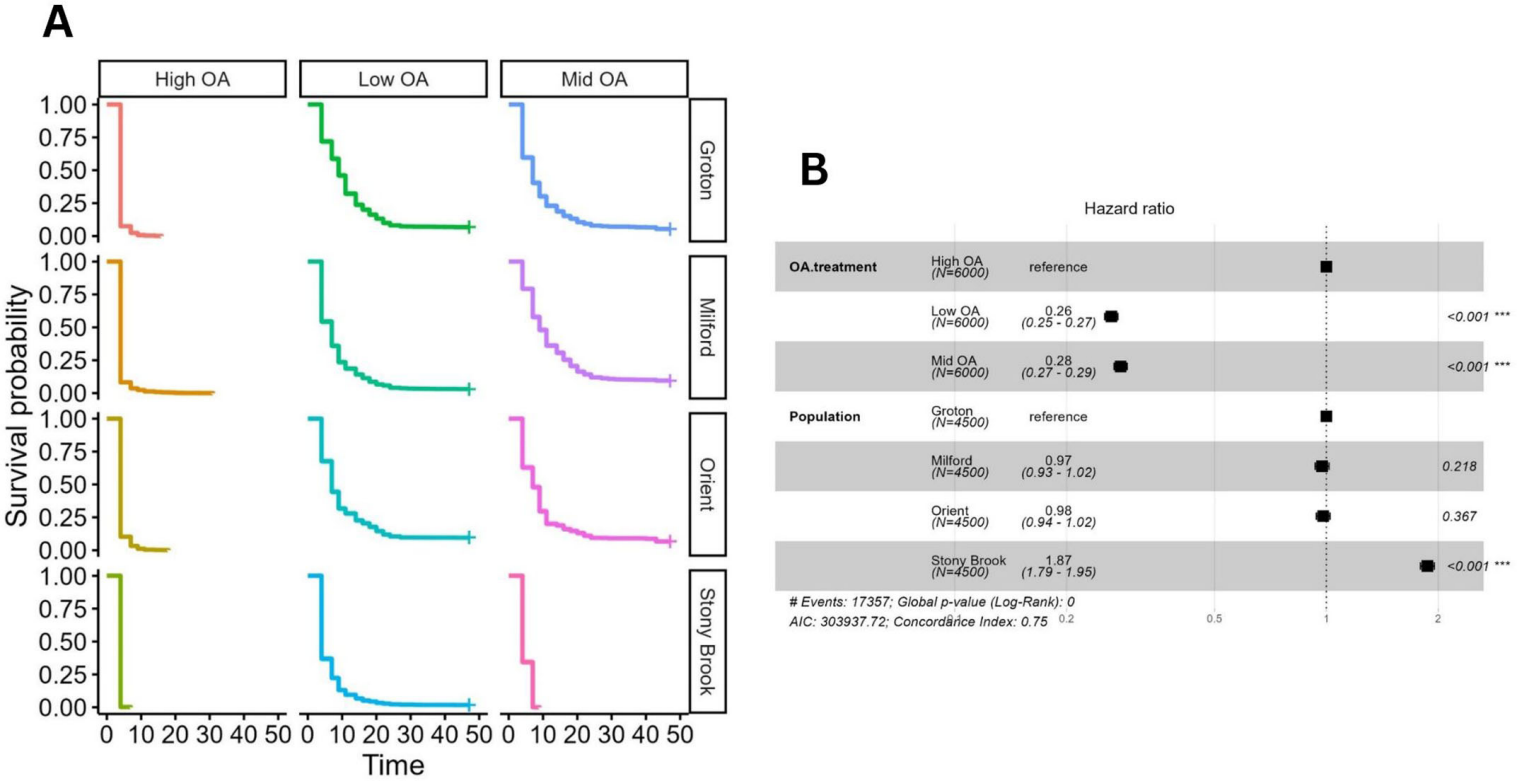
**Survivorship of larvae among populations and OA treatments.** (A) The probability of survivorship for larvae from each of the four test populations under each of the three test conditions. Test populations were Groton (G), Milford (M), Orient (O) and Stony Brook (S). The three OA treatments were: low OA (pH 7.9, Ω_ar_ 1.53, pCO_2_ 514.50), mid-OA (pH 7.57, Ω_ar_ 0.77, pCO_2_ 1167.13) high-OA (pH 7.26, Ω_ar_ 0.39, pCO_2_ 2479.74) (Table 3). No larvae survived to metamorphosis in the high-OA treatment, but there was a difference among populations in length of larval survival. Larvae survived to metamorphosis in the mid-OA treatment for all populations except Stony Brook. (B) The hazard ratio compares the rate of an event happening between two or more treatments. In this case, overall, the rate of death for the mid-OA treatment was 0.28 times less than that of the high-OA treatment (and was significant). The rate of death for larvae from Stony Brook (S) was 1.87 times greater than for larve from the Groton (reference) population (significant). Error bars are hidden by markers.

The effect of OA on survivorship of larvae was different among populations (Fig. 2). Both population (*P*=1.88e-238) and OA (*P*=0.000e+00) had highly significant effects on survivorship, and there was a significant population×OA interaction (*P*=9.78e-263; Table 2, Fig. 3), with populations responding differently to OA. Under the most extreme conditions (high OA) no larvae from any of the four populations survived to the end of the experiment, but larvae from Milford survived much longer than those from other populations. Larvae from the Stony Brook population had the lowest probability of survivorship; survivorship declined with increased OA and these larvae survived only in the low-OA treatment. Survivorship of blue mussel larvae from both Orient and Groton was not affected by mid- and low-OA conditions. Highest survivorship was for animals from Milford in the mid-OA treatment, which had much higher survivorship than those grown under low-OA conditions.

**Table 2.**
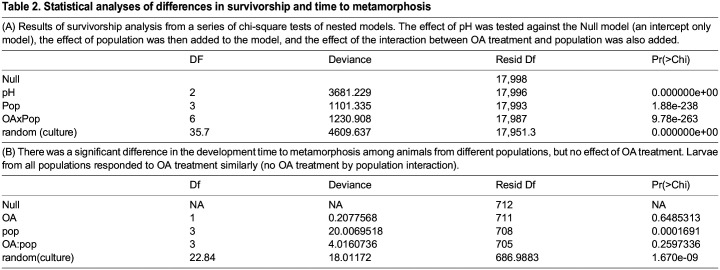
Statistical analyses of differences in survivorship and time to metamorphosis

**Table 3.**
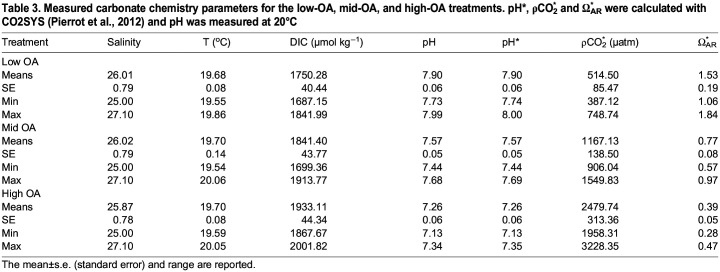
Measured carbonate chemistry parameters for the low-OA, mid-OA, and high-OA treatments. pH*, ρCO_2_^*^ and Ω_AR_^*^ were calculated with CO2SYS ( **Pierrot et al., 2012** **) and pH was measured at 20°C**

**Fig. 4.**
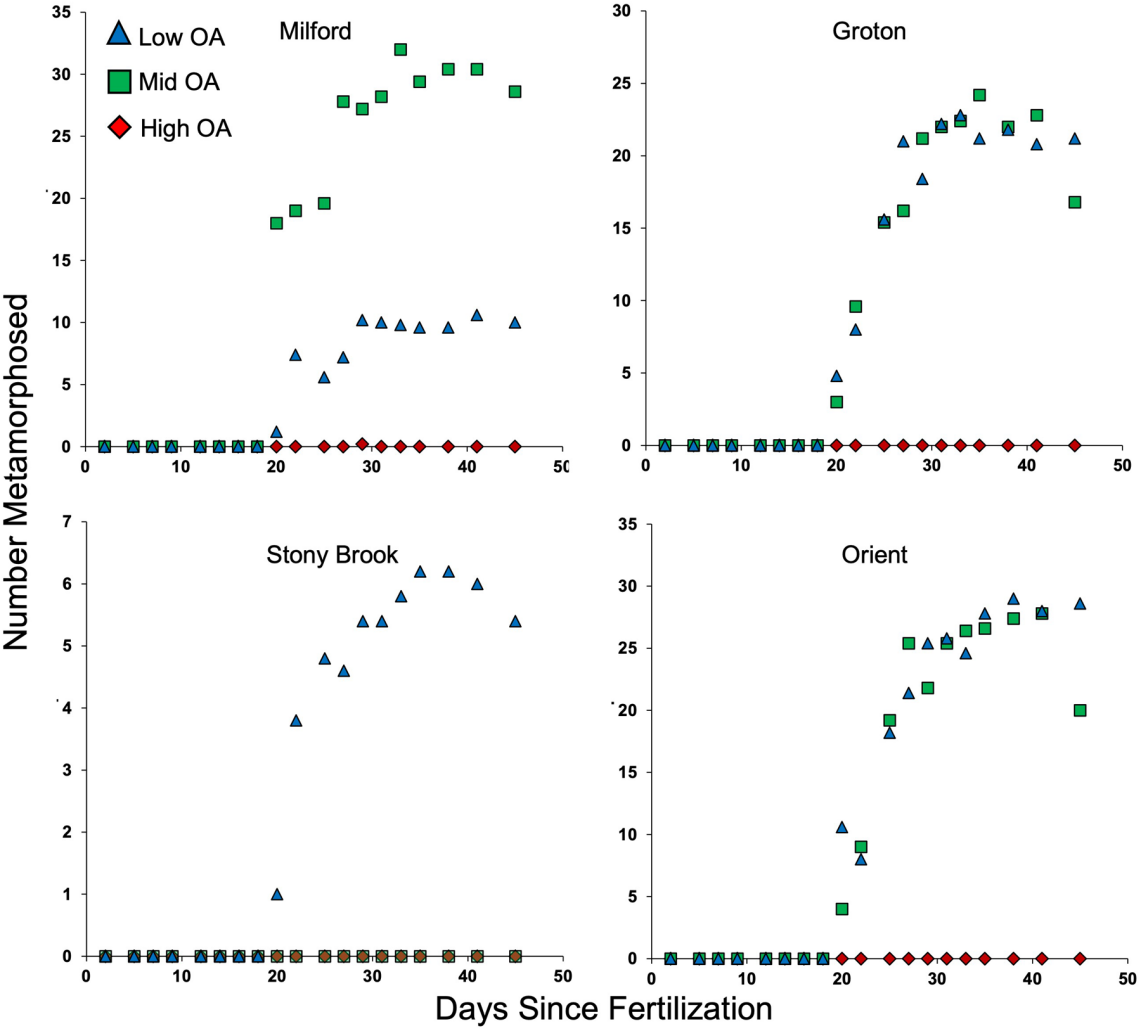
**Cumulative numbers of larvae that metamorphosed for the four test populations reared under each of the three OA conditions.** Shown are means of five replicates for each population. Low-OA (pH 7.9, Ω_ar_ 1.53, pCO_2_ 514.50), mid-OA (pH 7.57, Ω_ar_ 0.77, pCO_2_ 1167.13) high OA (pH 7.26, Ω_ar_ 0.39, pCO_2_ 2479.74).

**Fig. 5.**
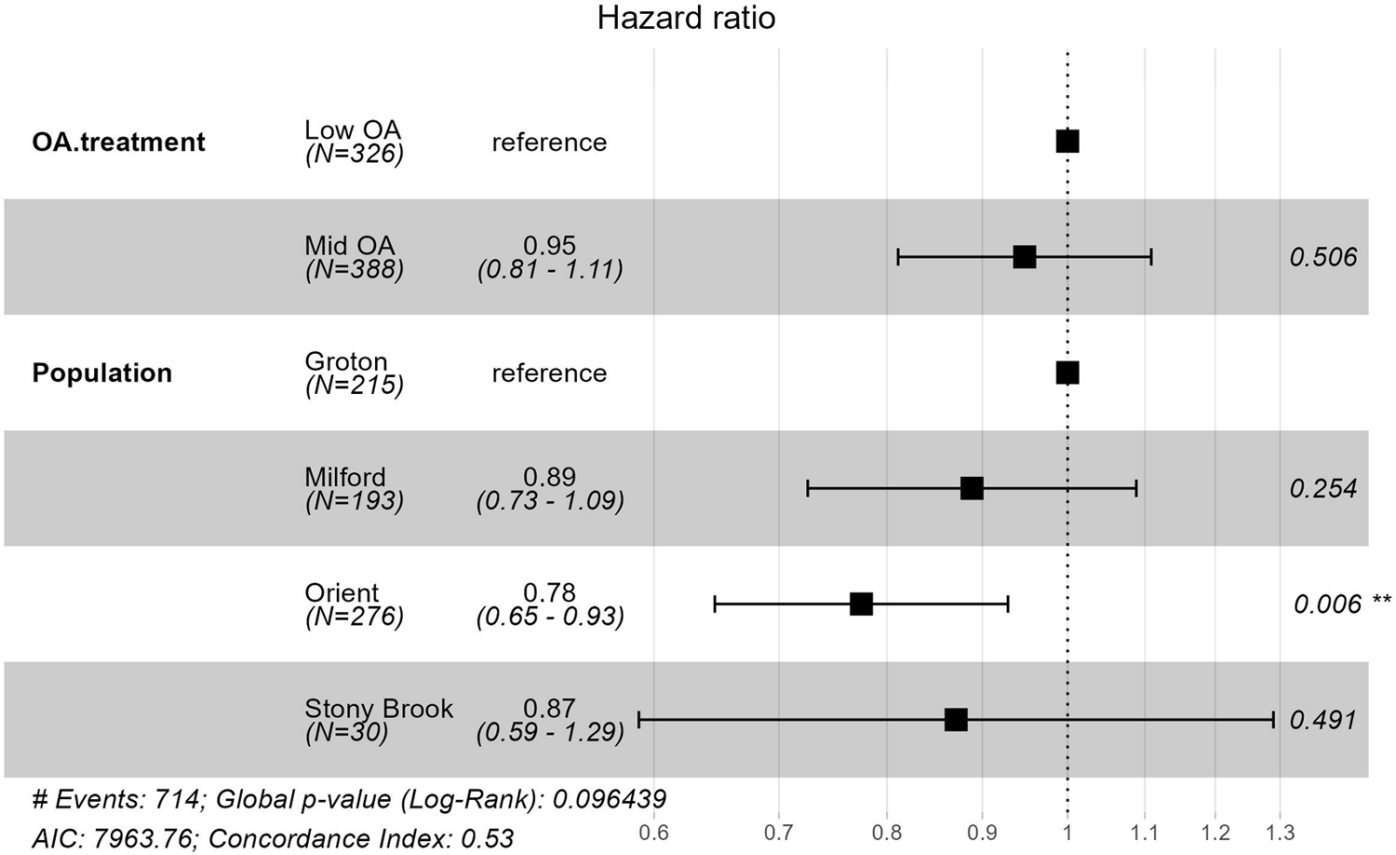
**The hazard ratio compares the rate of an event happening between two or more treatments.** The rate of metamorphosis under low-OA conditions was 1.05 times faster than that of mid-OA, but not significantly different. The rate of metamorphosis for larvae from Orient (O) was 0.78 times slower than that of larvae from Groton (G), and this difference was significant.

As observed for survivorship, there was a significant difference among populations in the rate of development as measured by time to metamorphosis (*P*=0.0002; Fig. 4 and 5, Table 2). Larvae from Groton had the fastest development, significantly faster than those from Orient, which were the slowest to reach metamorphosis. Contrary to survivorship, there was no effect of OA treatment on development time (*P*=0.6485), or OA treatment by population interaction (*P*=0.2597).

## DISCUSSION

We found striking differences in robustness to OA conditions for blue mussels from the four different populations sampled from locations within Long Island Sound. For all populations, there was no survivorship to metamorphosis in the high-OA treatment, the most extreme OA condition tested. We did find differences in survivorship among populations for the mid-OA and low-OA conditions. Mussels from both Orient and Groton, the two easternmost sites, where water conditions presently are more similar to oceanic conditions. Mussels at these sites rarely experience the OA conditions tested in our experiments (Table 1), but were robust to lower pH, lower Ω_ar_ and higher pCO_2_. There was no difference in survivorship between mid-OA and low-OA conditions for these two populations. For the other two populations, Stony Brook and Milford, we did see an effect of OA on survivorship, but not in the same direction. Larvae from Stony Brook showed the predicted response, and had the greatest sensitivity to OA; only larvae under the lowest-OA condition survived. Larvae from Milford, less than 50 km north of Stony Brook across Long Island Sound, had higher survivorship under mid-OA conditions than low-OA conditions, and thus appeared better able to cope with OA. Both Stony Brook and Milford mussels regularly experience seasonal OA conditions as severe as those tested in our experiment. For the Milford population, these results may indicate local adaptation to OA conditions. Overall, our results suggest that there is capacity for adaptation to OA, and we already see differences among populations in robustness to OA.

Contrary to other studies and general predictions (e.g. Talmage and Gobler, 2010; Meseck et al., 2021), there was no effect of OA on development time for any population; however, there was a difference in development time among populations. Although larvae from Groton and Orient were similarly robust in terms of survivorship to OA above the most severe conditions tested, they differed significantly in development time. Larvae from Groton developed faster than those from other populations, while larvae from the other eastern-most population, Orient, developed slower than others. These results suggest that genetic differences may underlie differences in response among populations, even those that are relatively close geographically, but any such differences would need to be tested with further research.

Our results demonstrate that the conclusions drawn from a given experimental study for a species may depend on the population or source stock used. Contrary to predictions that animals currently experiencing degraded habitats and low pH conditions would be more robust to OA, the two populations experiencing near oceanic conditions were the most robust to the tested OA conditions. At the same time, larvae from the Milford population had highest survivorship in the mid-OA treatment, and actually had lower survivorship under the low-OA (higher pH) treatment. These results, and the differences among populations in development time, suggest that aspects of our findings may be a result of genetic differences among populations and potentially local adaptation; however, this will require further study.

Prior research on blue mussels based on allozyme analyses found genetic differences among populations in eastern Long Island Sound (Hilbish, 1985; Koehn and Hilbish, 1987; Koehn, 1991). Eastern most populations had the same allele frequencies as open-coastal populations, but populations to the west (less than halfway between our more western test populations and those in the east), had different allozyme frequencies (Koehn and Hilbish, 1987; Koehn, 1991). The authors explained these patterns as evidence for selection for low salinity tolerance. Using demographic data, Hilbish (1985) suggested that selection for low-salinity tolerance was not at the larval stage, but was the result of differential juvenile survivorship. Therefore, a variety of environmental conditions may be affecting blue mussels at different stages during the life cycle. Determining whether there are differences in robustness to increasing OA conditions across life stages, and genetic potential for adaptation to OA, will require long-term experiments where individuals are exposed to increasing OA conditions across those life stage transitions.

Our findings have significant implications for experiments testing the effects of OA conditions on marine animals in general, illustrating the importance of testing individuals from multiple populations to assess the capacity for adaptation and response to this (and other) environmental challenges. Focusing on individuals from one site, as is typical for most studies of the effects of OA, is unlikely to capture the full potential of a species’ response, and could result in misleading conclusions. Our results are contrary to predictions that animals exposed to coastal OA conditions, or other environmental challenges, will be more robust to OA. For blue mussels, our results suggest that some populations are already robust to increased OA conditions, and they have the potential to adapt to OA as environments continue to change.

## MATERIALS AND METHODS

### Source populations

A minimum of 50 adult blue mussels (*M. edulis*, 30–66 mm maximum shell length) were collected from each of four populations around Long Island Sound (LIS). Populations were located in the east (Groton, G; Orient Point, O), and central parts (Milford, M, Stony Brook, S) on both the north and south shores (Fig. 1), where mussels experience very different environments. Historical carbonate chemistry for LIS is sparse, therefore, to understand the carbonate chemistry experienced by each population, monthly water samples were taken from each source location from July to January to understand seasonal and locational differences in water chemistry. In eastern LIS blue mussels experience near oceanic conditions with Ω_AR_ as high as 5.71, and to the west more estuarine and eutrophic environmental conditions exist (lower salinity, lower pH, and Ω_AR_<1.0, Table 1).

### OA Experimental conditions

An OA experimental system at the Northeast Fisheries Science Center in Milford CT was used for holding and conditioning adult mussels and for the larval experiments. Seawater for adults and larvae was pumped from Milford Harbor (41° 12′ 42.46″ N, 73° 3′ 7.75″ W). Adults were conditioned for spawning in a flow-through system, which is thoroughly described elsewhere (Perry et al., 2015; Meseck et al., 2021). Briefly, compressed air was scrubbed using a molecular-sieve CO_2_ adsorber (Puregas) down to <10 ppm. Scrubbed air and carbon dioxide were then mixed using mass-flow controllers (Aalborg Instruments and Controls, Orangeburg, NY, USA) with each treatment bubbled into PVC columns to produce CO_2_ enriched seawater. The system was designed to produce three different OA treatments (low-OA, mid-OA, and high-OA) following best experimental practices and data reporting guidelines established by EPOCA (Riebesell et al., 2011). Enriched air-CO_2_ was also constantly bubbled into each corresponding OA treatment container (Table 3). Larval experiments were conducted in a static OA system with the same mass-flow controllers and air-CO_2_ mixture used in the flow-through system. Filtered seawater (0.35 µm) was pre-equilibrated for 24 h prior to use, with water replacement in replicates every Monday, Wednesday, and Friday. During the experiment, the air-CO_2_ mixture for each treatment was constantly bubbled into each replicate bin (five bins per treatment, three treatments) to maintain the three OA treatment levels (Table 3). From the flow-through system, water samples were taken weekly, but in the static system (larval rearing), water samples were taken before and after the water was exchanged in each replicate bin three times a week. Samples were taken in dark polypropylene bottles (500 ml) from each tank and immediately analyzed for dissolved inorganic carbon (DIC) and pH. A YSI probe (model 556, Yellowspring, OH, USA) was used to record temperature and salinity at the time of sample collection. Measurements of pH were determined at 20°C spectrophotometrically (CARY 100) with m-cresol purple following the protocol outlined in the Guide to Best Practices for Ocean CO_2_ Measurements (Dickson et al., 2007). TRIS buffer obtained from the Dickson Laboratory (Scripps Institute, San Diego, CA, USA) were analyzed with each run to ensure accuracy (±0.0014). DIC measurements were analyzed using an Apollo SciTech's DIC Analyzer. Internal standards were used to calibrate the instrument and Certified Reference Material from the Dickson Laboratory was used to assure accuracy (±2.7 μmol kg^−1^). Precision of the carbonate chemistry measurements was confirmed by an international inter-laboratory comparison exercise with the laboratory being within 0.5% of the assigned values (Bockmon and Dickson, 2015). DIC and pH were used to back-calculate the saturation state of aragonite (Ω_ar_) and the pCO_2_ of each treatment using CO2SYS (Pierrot et al., 2012) with the following constants: K1, K2 from Mehrbach et al. (1973) refit by Dickson and Millero (1987); K hydrogen sulfate from Dickson et al. (1990); and total Boron from Uppström (1974).

Three OA test conditions, low-OA (pH 7.9, Ω_ar_ 1.53, pCO2 514.50), mid-OA (pH 7.57, Ω_ar_ 0.77, pCO2 1167.13) and high-OA (pH 7.26, Ω_ar_ 0.39, pCO2 2479.74), were produced (Table 3).

### Experimental details

Adult mussels from each source population were kept separately in screened cages in the flow-through system with natural plankton and supplemented daily with cultured microalgae (*Chaetoceros* sp.). Individuals from each population were divided equally among the three OA treatments and conditioned for 4 weeks. Prior to spawning, mussel shells were scrubbed to remove all attached organisms and then placed in 0.35 µm-filtered seawater. Spawning was induced by thermal shock alternating warm (up to 20°C) and cool (15°C) seawater until spawning was induced. In some cases, a male was dissected and sperm (from that population and OA treatment) was added to the water to induce spawning, and some animals were also injected with 0.5 ml of 2 M potassium chloride (KCl) to induce spawning (Strathmann, 1987). Spawning individuals were isolated in filtered sea water to keep gametes from mixing inadvertently.

The eggs from all females in a population and OA treatment combination were pooled, and then pooled sperm from males in that same population and OA treatment combination was used to fertilize eggs. Embryos for each population/OA treatment combination were placed in buckets with 15 L 0.35 µm filtered seawater (19°C, salinity 26) pre-conditioned to target OA condition, and allowed to develop for 48 h without food (larvae do not feed until the reach D stage). Two days after fertilization, larvae from each population and OA treatment level were allocated to replicate beakers (*n*=5).

### Larval experimental design

Forty-eight hours post-fertilization larvae were reared at 300 individuals l^−1^ in 0.35 µm-filtered sea water, fed 40,000 cells ml^−1^ of an equal mixture of *Tisochrysis lutea* and *Pavlova lutheri* (strain MONO). We used a factorial design with larvae from four populations×three OA treatments; each treatment was replicated five times, for a total of 60 larval cultures. Each replicate culture was held in a 1-L beaker with two large-screened windows (50 µm mesh size), allowing water and microalgal food to readily pass through. One beaker from each population was held in each replicate bin (10 L, *n*=5) for each OA treatment. Bins were held in a circulating, temperature-controlled bath, and distributed so that no two replicates of the same treatment were adjacent to each other. When bins were cleaned and replicate beakers replaced (three times per week), survivorship and the number of individuals that metamorphosed were determined, providing relatively high-resolution data. Animals were maintained in the static system until all individuals either died or metamorphosed.

### Data Analyses

Survivorship and time-to-metamorphosis data were analyzed with accelerated failure time (AFT) models (surviminer and survival packages, R version 3.6.1). These models accommodate censored data, in which the exact timing of the event (in this case, death or metamorphosis) happening in some subset of individuals is unknown after the experiment has ended. Data from this study were right censored. For the survivorship analysis, the date of death was unknown for individuals that metamorphosed, and their time of death was after the experiment had ended. For the time to metamorphosis analysis, those that died had an unknown true time to that developmental stage. The hazard ratio was also calculated, which allowed us to compare the slopes of survivorship and development curves. We fit models using exponential, log logistic, and Weibull distributions using the survival package in R software, and then used the Akaike's Information Criterion to choose the best model. We used population (S, G, O or M) and OA treatment (high, mid, low) as fixed factors, and the replicate culture as a random factor (individuals in the same culture are not independent). Because this experiment included both fixed and a random effect, we included a “frailty” term for replicate culture in the model for both survivorship and time to metamorphosis. Frailty models in survival analysis are analogous to mixed-model analysis of variance in measuring heterogeneity at the level of the frailty term.

## References

[BIO060479C1] Andersson, A. J., Mackenzie, F. T. and Bates, N. R. (2008). Life on the margin: implications of ocean acidification on Mg-calcite, high latitude and cold-water marine calcifiers. *Mar. Ecol. Prog. Ser*. 373, 265-274. http://www.jstor.org/stable/24872931 10.3354/meps07639

[BIO060479C2] Bayne, B. L. and Newell, R. C. (1983). Physiological energetics of marine molluscs. In *The Mollusca: Physiology, Part 1*, Vol. 4 (ed. K. M. Wilbur and A. S. M. Salenddin), pp. 407-515. London: Academic Press.

[BIO060479C3] Beyer, J., Green, N. W., Brooks, S., Allan, I. J., Ruus, A., Gomes, T., Bråte, I. L. and Schøyen, M. (2017). Blue mussels (*Mytilus edulis* spp.) as sentinel organisms in coastal pollution monitoring: a review. *Mar. Environmental Res.* 130, 338-365. 10.1016/j.marenvres.2017.07.024 28802590

[BIO060479C4] Bindoff, N. L., Cheung, W. W. L., Kairo, J. G., Arístegui, J., Guinder, V. A., Hallberg, R., Hilmi, N., Jiao, N., Karim, M. S. and Levin, L. et al. (2019). *Changing ocean, marine ecosystems, and dependent communities*. In IPCC special report on the ocean and cryosphere in a changing climate, Intergovernmental Panel on Climate Change (ed. H. O. Pörtner, D. C. Roberts, V. Masson-Delmotte, P. Zhai, M. Tignor, E. Poloczanska, K. Mintenbeck, A. Alegría, M. Nicolai, A. Okem, J. Petzold, B. Rama and N. M. Weyer). Geneva.

[BIO060479C5] Bockmon, E. E. and Dickson, A. G. (2015). An inter-laboratory comparison assessing the quality of seawater carbon dioxide measurements. *Mar. Chem.* 171, 36-43. 10.1016/j.marchem.2015.02.002

[BIO060479C6] Breitburg, D. L., Salisbury, J., Bernhard, J. M., Cai, W.-J., Dupont, S., Doney, S. C., Kroeker, K. J., Levin, L. A., Long, W. C., Milke, L. M. et al. (2015). And on top of all that… Coping with ocean acidification in the midst of many stressors. *Oceanography* 28, 48-61. 10.5670/oceanog.2015.31

[BIO060479C7] Clements, J. C. and Hunt, H. L. (2017). Effects of CO_2_-driven sediment acidification on infaunal marine bivalves: a synthesis. *Mar. Pollution Bull.* 117, 6-16. 10.1016/j.marpolbul.2017.01.053 28143647

[BIO060479C8] Coen, L. D., Brumbaugh, R. D., Bushek, D., Grizzle, R., Luckenbach, M. W., Posey, M. H., Powers, S. P. and Tolley, S. G. (2007). Ecosystem services related to oyster restoration. *Mar. Ecol. Prog. Ser.* 341, 303-307. 10.3354/meps341303

[BIO060479C9] Cooley, S. R., Rheuban, J. E., Hart, D. R., Luu, V., Glover, D. M., Hare, J. A. and Doney, S. C. (2015). An integrated assessment model for helping the United States sea scallop (*Placopecten magellanicus*) fishery plan ahead for ocean acidification and warming. *PLoS ONE* 10, e0124145. 10.1371/journal.pone.0124145 25945497 PMC4422659

[BIO060479C10] Cranford, P. J. (2019). Magnitude and extent of water clarification services provided by bivalve suspension feeding. In *Goods and Services of Marine Bivalves* (ed. A. C. Smaal, J. G. Ferreira, J. Grant, J. K. Petersen and Ø. Strand), pp. 119-141. Cham: Springer International Publishing.

[BIO060479C11] Cranford, P. J., Strain, P. M., Dowd, M., Hargrave, B. T., Grant, J. and Archambault, M.-C. (2007). Influence of mussel aquaculture on nitrogen dynamics in a nutrient enriched coastal embayment. *Mar. Ecol. Prog. Ser.* 347, 61-78. 10.3354/meps06997

[BIO060479C12] Dickson, A. G., Wesolowski, D. J., Palmer, D. A. and Mesmer, R. E. (1990). Dissociation constant of bisulfate ion in aqueous sodium chloride solutions to 250. degree. *C*. *J. Phys. Chem* 94, 7978-7985. 10.1021/j100383a042

[BIO060479C13] Dickson, A. G. and Millero, F. J. (1987). A comparison of the equilibrium constants for the dissociation of carbonic acid in seawater media. *Deep Sea Res. A. Oceanograph. Res. Papers* 34, 1733-1743. 10.1016/0198-0149(87)90021-5

[BIO060479C14] Dickson, A. G., Sabine, C. L. and Christian, J. R. (2007). *Guide to Best Practices for Ocean CO2 Measurements*. Report. *North Pacific Marine Science Organization*.

[BIO060479C15] Dupont, S., Lundve, B. and Thorndyke, M. (2010). Near future ocean acidification increases growth rate of the lecithotrophic larvae and juveniles of the sea star *Crossaster papposus*. *J. Exp. Zool. B: Mol. Dev. Evol.* 314B, 382-389. 10.1002/jez.b.21342 20309996

[BIO060479C16] Ekstrom, J. A., Suatoni, L., Cooley, S. R., Pendleton, L. H., Waldbusser, G. G., Cinner, J. E., Ritter, J., Langdon, C., van Hooidonk, R., Gledhill, D. et al. (2015). Vulnerability and adaptation of US shellfisheries to ocean acidification. *Nat. Clim. Change* 5, 207-214. 10.1038/nclimate2508

[BIO060479C17] Enderlein, P. and Wahl, M. (2004). Dominance of blue mussels versus consumer-mediated enhancement of benthic diversity. *J. Sea Res.* 51, 145-155. 10.1016/j.seares.2003.05.006

[BIO060479C18] Farrington, J. W., Tripp, B. W., Tanabe, S., Subramanian, A., Sericano, J. L., Wade, T. L., Knap, A. H. and Edward, D. (2016). Goldberg‘s proposal of “the mussel watch”: Reflections after 40 years. *Mar. Pollution Bull.* 110, 501-510. 10.1016/j.marpolbul.2016.05.074 27339743

[BIO060479C19] Feely, R. A., Alin, S. R., Newton, J., Sabine, C. L., Warner, M., Devol, A., Krembs, C. and Maloy, C. (2010). The combined effects of ocean acidification, mixing, and respiration on pH and carbonate saturation in an urbanized estuary. *Estuar. Coast. Shelf Sci.* 88, 442-449. 10.1016/j.ecss.2010.05.004

[BIO060479C21] Gazeau, F., Parker, L. M., Comeau, S., Gattuso, J.-P., O‘Connor, W. A., Martin, S., Pörtner, H.-O. and Ross, P. M. (2013). Impacts of ocean acidification on marine shelled molluscs. *Mar. Bio.* 160, 2207-2245. 10.1007/s00227-013-2219-3

[BIO060479C22] Gledhill, D. K., White, M. M., Salisbury, J., Thomas, H., Mlsna, I., Liebman, M., Mook, B., Grear, J., Candelmo, A. C., Chambers, R. C. et al. (2015). Ocean and coastal acidification off New England and Nova Scotia. *Oceanography* 28, 182-197. 10.5670/oceanog.2015.41

[BIO060479C23] Goldberg, E. D. (1975). The mussel watch — A first step in global marine monitoring. *Mar. Pollut. Bull.* 6, 111. 10.1016/0025-326X(75)90271-4

[BIO060479C25] Gooding, R. A., Harley, C. D. G. and Tang, E. (2009). Elevated water temperature and carbon dioxide concentration increase the growth of a keystone echinoderm. *Proc. Natl. Acad. Sci. U.S.A.* 106, 9316-9321. 10.1073/pnas.0811143106 19470464 PMC2695056

[BIO060479C26] Grear, J. S., O‘Leary, C. A., Nye, J. A., Tettelbach, S. T. and Gobler, C. J. (2020). Effects of coastal acidification on North Atlantic bivalves: interpreting laboratory responses in the context of in situ populations. *Mar. Ecol. Prog. Ser.* 633, 89-104. 10.3354/meps13140 34121786 PMC8193825

[BIO060479C27] Harvey, B. P., McKeown, N. J., Rastrick, S. P. S., Bertolini, C., Foggo, A., Graham, H., Hall-Spencer, J. M., Milazzo, J., Shaw, P. W., Small, D. P. et al. (2016). Individual and population-level responses to ocean acidification. *Sci. Rep.* 6, 20194. 10.1038/srep20194 26822220 PMC4731747

[BIO060479C28] Hilbish, T. J. (1985). Demographic and temporal structure of an allele frequency cline in the mussel *Mytilus edulis*. *Mar. Biol.* 86, 163-171. 10.1007/BF00399023

[BIO060479C29] Hunt, C. W., Salisbury, J. E. and Vandemark, D. (2022). Controls on buffering and coastal acidification in a temperate estuary. *Limnol. Oceanogr.* 67, 1328-1342. 10.1002/lno.12085

[BIO060479C30] Kelly, M. W. and Hofmann, G. E. (2013). Adaptation and the physiology of ocean acidification. *Funct. Ecol.* 27, 980-990. 10.1111/j.1365-2435.2012.02061.x

[BIO060479C31] Kleypas, J. A., Feely, R. A., Fabry, V. J., Langdon, C., Sabine, C. L. and Robbins, L. L. (2006). Impacts of Ocean Acidification on Coral Reefs and Other Marine Calcifiers: A Guide for Future Research, report of a workshop held 18–20 April 2005, St. Petersburg, FL, sponsored by NSF, NOAA, and the U.S. Geological Survey, 88 pp.

[BIO060479C32] Koehn, R. K. (1991). The genetics and taxonomy of species in the genus *Mytilus*. *Aquaculture* 94, 125-145. 10.1016/0044-8486(91)90114-M

[BIO060479C33] Koehn, R. K. and Hilbish, T. J. (1987). The adaptive importance of genetic variation. *Am. Sci.* 75, 134-141.

[BIO060479C34] Kroeker, K. J., Kordas, R. L., Crim, R., Hendriks, I. E., Ramajo, L., Singh, G. S., Duarte, C. M. and Gattuso, J.-P. (2013). Impacts of ocean acidification on marine organisms: quantifying sensitivities and interaction with warming. *Glob. Change Biol.* 19, 1884-1896. 10.1111/gcb.12179 PMC3664023 23505245

[BIO060479C35] Mehrbach, C., Culberson, C. H., Hawley, J. E. and Pytkowicx, R. M. (1973). Measurement of the apparent dissociation constants of carbonic acid in seawater at atmospheric pressure 1. *Limnol. Oceanogr.* 18, 897-907. 10.4319/lo.1973.18.6.0897

[BIO060479C36] Melzner, F., Gutowska, M. A., Langenbuch, M., Dupont, S., Lucassen, M., Thorndyke, M. C., Bleich, M. and Pörtner, H.-O. (2009). Physiological basis for high CO_2_ tolerance in marine ectothermic animals: pre-adaptation through lifestyle and ontogeny? *Biogeosciences* 6, 2313-2331. 10.5194/bg-6-2313-2009

[BIO060479C37] Menge, B. A. (2000). Top-down and bottom-up community regulation in marine rocky intertidal habitats. *J. Exp. Mar. Biol. Ecol.* 250, 257-289. 10.1016/S0022-0981(00)00200-8 10969172

[BIO060479C38] Meseck, S. L., Mercaldo-Allen, R., Clark, P., Kuropat, C., Redman, D., Veilleux, D. and Milke, L. (2021). Effects of ocean acidification on larval Atlantic surfclam (*Spisula solidissima*) from Long Island Sound in Connecticut. *Fish. Bull* 119, 66-76. 10.7755/FB.119.1.8

[BIO060479C39] Munday, P. L., Warner, R. R., Monro, K., Pandolfi, J. M. and Marshall, D. J. (2013). Predicting evolutionary responses to climate change in the sea. *Ecol. Lett.* 16, 1488-1500. 10.1111/ele.12185 24119205

[BIO060479C40] Nielsen, P., Cranford, P. J., Maar, M. and Petersen, J. K. (2016). Magnitude, spatial scale and optimization of ecosystem services from a nutrient extraction mussel farm in the eutrophic Skive Fjord, Denmark. *Aquac. Environ. Interact* 8, 311-329. 10.3354/aei00175

[BIO060479C42] Perry, D. M., Redman, D. H., Widman, J. C., Meseck, S., King, A. and Pereira, J. J. (2015). Effect of ocean acidification on growth and otolith condition of juvenile scup, *Stenotomus chrysops*. *Ecol. Evol* 5, 4187-4196. 10.1002/ece3.1678 26442471 PMC4588644

[BIO060479C43] Pierrot, D., Lewis, E. and Wallace, D. W. R. (2012). MS Excel program developed for CO2 system calculations. U.S. Department of Energy, Oak Ridge National Laboratory, Carbon Dioxide Information Analysis Center, ORNL/CDIAC-105a, Oak Ridge, Tennessee.

[BIO060479C44] Pistevos, J. C. A., Calosi, P., Widdicombe, S. and Bishop, J. D. D. (2011). Will variation among genetic individuals influence species responses to global climate change? *Oikos* 120, 675-689. 10.1111/j.1600-0706.2010.19470.x

[BIO060479C45] Pörtner, H. O. and Farrell, A. P. (2008). Ecology physiology and climate change. *Science* 322, 690-692. 10.1126/science.1163156 18974339

[BIO060479C47] Reusch, T. B. H. (2014). Climate change in the oceans: evolutionary versus phenotypically plastic responses of marine animals and plants. *Evol. App.* 7, 104-122. 10.1111/eva.12109 PMC3894901 24454551

[BIO060479C48] Rheuban, J. E., Doney, S. C., Cooley, S. R. and Hart, D. R. (2018). Projected impacts of future climate change, ocean acidification, and management on the US Atlantic sea scallop (*Placopecten magellanicus*) fishery. *PLoS ONE* 13, e0203536. 10.1371/journal.pone.0203536 30240399 PMC6150507

[BIO060479C49] Riebesell, U., Fabry, V. J., Hansson, L. and Gattuso, J.-P. (2011). *Guide to best practices for ocean acidification research and data reporting. [reprinted edition including erratum]*. Report. *Publications Office of the European Union*.

[BIO060479C50] Salisbury, J., Green, M., Hunt, C. and Campbell, J. (2008). Coastal acidification by rivers: a threat to shellfish? *Eos, Transactions American Geophysical Union* 89, 513-513. 10.1029/2008EO500001

[BIO060479C51] Schoener, T. W. (2011). The newest synthesis: understanding the interplay of evolutionary and ecological dynamics. *Science (New York, N.Y.)* 331, 426-429. 10.1126/science.1193954 21273479

[BIO060479C52] Siedlecki, S. A., Salisbury, J., Gledhill, D. K., Bastidas, C., Meseck, S., McGarry, K., Hunt, C. W., Alexander, M., Lavoie, D., Wang, Z. A. et al. (2021). Projecting ocean acidification impacts for the Gulf of Maine to 2050: New tools and expectations. *Elem. Sci. Anth.* 9, 00062. 10.1525/elementa.2020.00062

[BIO060479C53] Smaal, A. C., Ferreira, J. G., Grant, J., Petersen, J. K. and Strand, Ø. (2019). *Goods and Services of Marine Bivalves*, p. 584. UK: Springer.

[BIO060479C54] Somero, G. N., Beers, J. M., Chan, F., Hill, T. M., Klinger, T. and Litvin, S. Y. (2016). What changes in the carbonate system, oxygen, and temperature portend for the Northeastern Pacific Ocean: a physiological perspective. *Bioscience* 66, 14-26. 10.1093/biosci/biv162

[BIO060479C55] Sorte, C. J. B., Davidson, V. E., Franklin, M. C., Benes, K. M., Doellman, M. M., Etter, R. J., Hannigan, R. E., Lubchenco, J. and Menge, B. A. (2017). Long-term declines in an intertidal foundation species parallel shifts in community composition. *Glob. Change Biol.* 23, 341-352. 10.1111/gcb.13425 27411169

[BIO060479C56] Strathmann, M. F. (1987). *Reproduction and Development of Marine Invertebrates of the Northern Pacific Coast: Data and Methods for the Study of Eggs, Embryos, and Larvae*. University of Washington Press.

[BIO060479C57] Sunday, J. M., Crim, R. N., Harley, C. D. G. and Hart, M. W. (2011). Quantifying rates of evolutionary adaptation in response to ocean acidification. *PLoS ONE* 6, e22881. 10.1371/journal.pone.0022881 21857962 PMC3153472

[BIO060479C58] Talmage, S. C. and Gobler, C. J. (2010). Effects of past, present, and future ocean carbon dioxide concentrations on the growth and survival of larval shellfish. *Proc. Natl. Acad. Sci. USA* 107, 17246-17251. 10.1073/pnas.0913804107 20855590 PMC2951451

[BIO060479C59] Thomsen, J., Stapp, L. S., Haynert, K., Schade, H., Danelli, M., Lannig, G., Wegner, K. M. and Melzner, F. (2017). Naturally acidified habitat selects for ocean acidification–tolerant mussels. *Sci. Adv.* 3, e1602411. 10.1126/sciadv.1602411 28508039 PMC5406135

[BIO060479C60] Uppström, L. R. (1974). The boron/chlorinity ratio of deep-sea water from the Pacific Ocean. *Deep-Sea Res. Oceanogr. Abstr.* 21, 161-162. 10.1016/0011-7471(74)90074-6

[BIO060479C61] van der Schatte Olivier, A., Jones, L., Vay, L. L., Christie, M., Wilson, J. and Malham, S. K. (2020). A global review of the ecosystem services provided by bivalve aquaculture. *Reviews in Aquaculture* 12, 3-25. 10.1111/raq.12301

[BIO060479C62] Vargas, C. A., Cuevas, L. A., Broitman, B. R., San Martin, V. A., Lagos, N. A., Gaitán-Espitia, J. D. and Dupont, S. (2022). Upper environmental pCO_2_ drives sensitivity to ocean acidification in marine invertebrates. *Nat. Clim. Change* 12, 200-207. 10.1038/s41558-021-01269-2

[BIO060479C63] Waldbusser, G. G. and Salisbury, J. E. (2014). Ocean acidification in the coastal zone from an organism‘s perspective: multiple system parameters, frequency domains, and habitats. *Ann. Rev. Mar. Sci.* 6, 221-247. 10.1146/annurev-marine-121211-172238 23987912

[BIO060479C64] Waldbusser, G. G., Hales, B., Langdon, C. J., Haley, B. A., Schrader, P., Brunner, E. L., Gray, M. W., Miller, C. A. and Gimenez, I. (2015). Saturation-state sensitivity of marine bivalve larvae to ocean acidification. *Nat. Clim. Change* 5, 273-280. 10.1038/nclimate2479 PMC4465621 26061095

[BIO060479C65] Wood, H. L., Sundell, K., Almroth, B. C., Sköld, H. N. and Eriksson, S. P. (2016). Population-dependent effects of ocean acidification. *Proc. R. Soc. B* 283, 20160163. 10.1098/rspb.2016.0163 PMC4843657 27053741

[BIO060479C66] Zeebe, R. E. (2012). History of seawater carbonate chemistry, atmospheric CO_2_, and ocean acidification. *Annu. Rev. Earth Planet. Sci.* 40, 141-165. 10.1146/annurev-earth-042711-105521

[BIO060479C67] Zeebe, R. E., Ridgwell, A. and Zachos, J. C. (2016). Anthropogenic carbon release rate unprecedented during the past 66 million years. *Nat. Geosci.* 9, 325-329. 10.1038/ngeo2681

